# Recreation in Relation to the Health of the People

**Published:** 1891-08-01

**Authors:** 


### Recreation in Relation to the Health of the People.

### VI.—IN THE COUNTRY.

It has been said—and said, we think, with considerable truth—that London has too large an amount of attention bestowed upon it by us English people, as compared with that which we vouchsafe to other towns and to the country. Just as some schoolmistresses treat the naughtiest child in the school, trying to keep it good by all sorts of little concessions, and offering it cakes and sugar-plums if it will only sit still and do its lessons, until it discovers that naughtiness pays far best, and trades upon that discovery, so do we behave to this unmanageable metropolis of ours, increasing that unmanageableness by our very efforts to cope with it. We see a crowd of people waiting to be amused, and we try to amuse them; we see them dragging down their own wages by overcrowding the labour market, and we try to raise those wages; we find them huddling together in overcrowded, filthy, and yet high-rented rooms, and we set our wits to work at the task of providing them with decent habitations. What is the result? Toil as we may, we can hardly keep pace with the work, much less overtake it; the naughty child becomes more tiresome and out-of-hand every day, and we sometimes feel inclined to throw up our hands in despair, and say, What is the good of it all?

It was the *Spectator*, we think, which, amid the chorus of praise which arose when Sir Edward Guinness's scheme for building model dwellings in London and Dublin was first made known, remarked that it could wish that less money had been devoted to London, and more to Dublin and other towns, on the ground that London was too much before the public—the working man's public—already. And if we think too exclusively of London with regard to house accommodation, so also do we think too exclusively of it in our efforts to provide recreation for the people. Those who watch the shifting to and fro—nay, it is practically all to and no fro—of our provincial and metropolitan populations, complain that a country town now possesses no attractions as compared with London. And if this be true of country towns, still more is it true of the real and unadulterated country. We remember reading, some time ago, a bitter complaint by a country clergyman to the effect that all his flock were drifting off to London—and what wonder? There they had water and gas laid on; there they had brightly-lighted and comparatively clean streets; there—and this brings us to the special point which we wish to make—there was always something going on—a play, a concert, a debate, a club-meeting—something to rest the tired mind and revive the drooping spirits.

Unless we wish, then—and surely nobody can wish—to see the country depopulated and the towns more than ever crowded into, it becomes of the utmost importance for those who believe in recreation to take care that it is not supplied in London alone, nor even only in towns, whatever their size, but that country people too shall have their share in it.

Of course, there are difficulties in the way. One difficulty is the want of money, and a serious difficulty it sometimes is, as those who see the straitened means of landowners and country clergymen nowadays are well aware. But why should not philanthropists feel that they have duties towards the country as well as towards the towns—that their duty, in fact, is to England as a whole, not to a certain bit of it which has forced itself into special notice? True, the founding of a new People's Palace in London is more dramatic, and attracts more attention than, let us say, the hunting out of a certain number of villages where suitable clubs and coffee-houses may be built; but it is not all philanthropists, surely, who care about nothing but making a grand *coup*; and we do sincerely believe that he who would do the latter would accomplish quite as useful work as he who did the former.

Then, again, with regard to workers. How many ladies there are who are eager to devote their lives to the service of others! But they all go into the towns, and seek work there. Why not try the country? Plenty of work might be found for them there, as many a country clergyman, single-handed and down-hearted, could tell them. He must needs have the distinctly spiritual wants of his people first and foremost in his mind; and it would be a great help to him, as well as to his people, to have someone at hand who would devote his or her time to making the village life bright and cheerful.

We are inclined to think, however, that country clergymen, as a rule, are not sufficiently awake to the necessity of providing amusement for their people. "We never have any evening entertainments here. I don't wish to bring the young people out at night," was lately said to the writer by the rector of a country parish. Apparently it never occurred to him to enquire whether the young people did not go out at night already; whether it was at all likely that they would stop at home, when the evening was their only time for amusing themselves; whether it would not be well, as things stood, to see that some at least of that amusement should be safe and wholesome. "Wherever shall we take our young ladies to?" cried some of the frequenters of the "Victoria Hall," when it was proposed to close it for the summer months; and this might well be the constant cry of the lads in many a country village. Is the public-house a proper place for Giles to take his "young lady" to? Or should he and she rather loiter about the village street, or go for a walk in the dark country lanes? The only other alternative is for poor Giles to do his courting under the eyes of his prospective father and mother-in-law, and perhaps a tribe of youngsters; and—well, perhaps the Giles of Utopia might be content with that, but we fear the Giles of every-day life will not.

And even without his "young lady," Giles wants amusing. In the summer, indeed, he is pretty well off, if he has a garden or an allotment to see to, though, even then, we think that a little attention might profitably be given to providing recreation for him in the shape of cricket and other games. A recreation ground, again, would be a great boon to many a country village, and one that must be comparatively easy to secure. But it is, of course, in the long winter evenings that recreation is specially needed. There are plenty of temptations for a lad whose time hangs heavy on his hands, even though he live in the country; and those who start village classes for singing, carving, or clay-modelling, or get up entertainments in which the village people themselves can take a share, may, we are sure, do much to keep the lads from wasting their money and besotting their minds at the public-house.

As to physical recreation, it should not of course take a large place where the class is composed, as in a village, of lads who have been at work in the fields all day; but we believe that a few minutes of musical drill would be not only popular, but very useful. It is said that the peculiar rolling gait of the agricultural labourer, though very uncouth in itself, is really an excellent instance of "adaptation to environment," and that it is this which enables him to beat all ordinary walkers hollow at a tramp across ploughed fields. That may be; but we do not see why he should also stoop and slouch, making himself into an old man when he should be yet in his prime; and we should like to see a few minutes' drill introduced into all evening schools, whether they be in town or country.

